# Analysis of human leukocyte antigen allele polymorphism in patients with non alcoholic fatty liver disease

**DOI:** 10.1097/MD.0000000000016704

**Published:** 2019-08-09

**Authors:** Azza Karrar, Siddharth Hariharan, Yousef Fazel, Ali Moosvi, Mohamad Houry, Zahra Younoszai, Thomas Jeffers, Li Zheng, Otgonsuren Munkhzul, Sharon Hunt, Fanny Monge, Zachary Goodman, Zobair M. Younossi

**Affiliations:** a Betty and Guy Beatty Center for Integrated Research, Inova Health System; b Center for Liver Diseases, Department of Medicine, Inova Fairfax Hospital, Falls Church, VA.

**Keywords:** HLA class I, HLA class II, NAFLD, NASH, steatohepatitis, steatosis

## Abstract

The human leukocyte antigen (HLA) genes may play a role in the pathogenesis of non-alcoholic fatty liver disease (NAFLD) and its progressive form, non-alcoholic steatohepatitis (NASH). The aim of this study was to assess the association of HLA class I and II alleles with NASH and its histological features.

Deoxyribonucleic acid (DNA) was extracted from 140 subjects (85 biopsy-proven NAFLD and 55 controls) and genotyped for HLA (-A, -B, -C, -DR1, -DR3, -DQ, and -DP). Liver biopsies were assessed for presence of NASH, degree of fibrosis and inflammation. Multivariate analysis was performed to assess associations between HLA genes and different histologic features of NAFLD.

Our data for HLA class I showed that HLA-C∗4 was associated with lower risk for histologic NASH and HLA-C∗6 was protective against portal fibrosis. Conversely, HLA-B∗27 was associated with high-grade hepatic steatosis, while HLA-A∗31 was associated with increased risk for advanced fibrosis. Among HLA class II alleles, HLA-DQA1∗01 was associated with lower risk for NASH while HLA-DRB1∗03 was associated with increased risk for NASH.

Our findings indicate that HLA class I and II gene polymorphism may be associated with susceptibility to NASH, fibrosis and other pathologic features and may be involved in the pathogenesis of NAFLD.

**What is already known about this subject?**

- Within the HLA class I Locus, HLA-B∗65 has been shown to be independently associated with the development of NAFLD.
- Within the HLA class II Locus, HLA-DQB1∗6:04 has been shown to be a risk factor for NALFD while HLA-DQB1∗03:02 has been shown to be protective against NALFD. HLA- DQ5 has been significantly associated with NAFLD.
- The information regarding the clinical significance of HLA antigens as an immunomodulatory molecule in NAFLD/NASH related fibrosis is limited.

**What does your study add?**

- Although no alleles within HLA class I were independently associated with NAFLD, HLA-B∗27 was independently associated with higher risk for high-grade steatosis. HLA-DRB1∗07 was independently associated with higher risk for NAFLD.
- HLA-C∗4 was found to be associated with lower risk for NASH NAFLD and protective against pericellular fibrosis, while HLA-C∗6 was independently protective against portal fibrosis.
- HLA-DQA1∗01 was independently associated with lower risk for NASH NAFLD and lower risk for pericellular and portal fibrosis, while HLA-DRB∗03 was independently associated with a higher risk for NASH NAFLD.

## 1 Introduction

Non-alcoholic fatty liver disease (NAFLD) is a common form of chronic liver disease. There is increasing evidence that NAFLD and its subtype non-alcoholic steatohepatitis (NASH) result from a complex interplay between environmental and host factors.^[1]^ In this context, NAFLD and NASH are the phenotypes that results from multiple pathogenic pathways. The role of genetic variables in the pathogenesis of NAFLD and NASH and its progression has been implicated. In this context, the human leukocyte antigen (HLA) is a genetic marker that may point to the host immune factors that play a role in the pathogenesis and progression of NAFLD. In fact, HLA-B∗65 has been shown to be independently associated with the development of NAFLD.^[2]^ Furthermore, HLA-B∗35 and -B∗40 have been linked to the development of cirrhosis in patients with alcoholic fatty liver disease.^[3–5]^ Within the HLA class II, HLA- DQ5 has been significantly associated with NAFLD.^[2]^ In contrast, HLA-DQB1∗6:04 has been shown to be a risk factor for NALFD while HLA-DQB1∗03:02 has been shown to be protective against NALFD.^[6]^

It is also important to note that HLA class I and II have been implicated in other liver diseases. In fact, a recent study suggested that HLA-DRB1∗03, ∗04, and ∗13 alleles may be involved in chronic hepatitis C virus (HCV) infection.^[7]^ The HLA-DRB1 locus was not only associated with HCV but also with Hepatitis B Virus (HBV) infection. In this context, a long term study showed that HLA-DRB1∗140101 was a potential risk factor to develop Hepatocellular Carcinoma (HCC) in patients with HBV.^[8]^ Therefore, the aim of this study was to assess the association between the HLA class I and II alleles (HLA-A, -B, -C, -DR, -DR3, -DQ, and -DP) with NAFLD and its subtype, NASH.

## 2 Methods

### 2.1 Study design: study cohort, blood and liver tissue collection

The final study cohort included 140 subjects who were prospectively enrolled after complete informed consent. Among the 140 subjects, 85 patients had biopsy-proven NAFLD, and 55 were healthy controls without liver disease. At the time of liver biopsy, blood samples, demographic and clinical data were collected. Patients with other liver diseases and/or evidence of excessive alcohol use (≥10 g/d) were excluded. The controls were subjects who were scheduled for living related kidney donations and all specimens were obtained after informed consent

From all samples, sera were separated and peripheral blood mononuclear cells (PBMCs) were snap-frozen at −80°C for subsequent deoxyribonucleic acid (DNA) extraction. The study was conducted after Institutional Review Board (IRB) approval at Inova Fairfax Hospital was obtained.

### 2.2 NAFLD diagnosis and histopathological assessment of liver biopsies

Liver tissues were initially fixed in formalin in preparation for sectioning and histopathologic evaluation. Liver biopsy sections were stained with hematoxylin and eosin (H&E) and Masson Trichrome for steatosis and fibrosis detection, respectively. All biopsies were evaluated by 1 hepatopathologist (ZG) for the degree of steatosis (0–3), portal inflammation (0–3), lymphoplasmacytic lobular inflammation (0–3), degenerative ballooning (0–2), Mallory-Denk bodies, apoptotic bodies, focal necrosis and interlobular pericellular fibrosis, portal fibrosis (0–3), and presence of bridging fibrosis and cirrhosis. NAFLD diagnosis is defined by more than 5% macrovesicular hepatic steatosis. NASH diagnosis included patients with hepatic steatosis and inflammation, together with hepatocyte injury with degenerative ballooning with or without Mallory-Denk bodies and/or pericellular fibrosis.^[9,10]^ Advanced histologic features are defined by stage ≥2. Mallory-Denk bodies, apoptotic bodies, and focal necrosis were scored as either present or absent.

### 2.3 HLA class I and class II genotyping

#### 2.3.1 DNA isolation fromPBMCs

DNA was isolated from 200 μL of PBMCs from patients and controls using QIAmp DNA isolation Kit (QIAGEN, MD) according to the manufacturer's instructions. The quality of extracted DNA was determined by spectrophotometric analysis. A260/A280 ratio of 1.6 to 2.0 was considered good quality. Integrity of Deoxyribonucleic Acid (DNA) was confirmed by agarose gel electrophoresis. The eluted DNA was stored at −20°C for long term storage.

#### 2.3.2 Polymerase chain reaction sequence-specific oligonucleotide (PCR-SSO)

**Figure 1:**
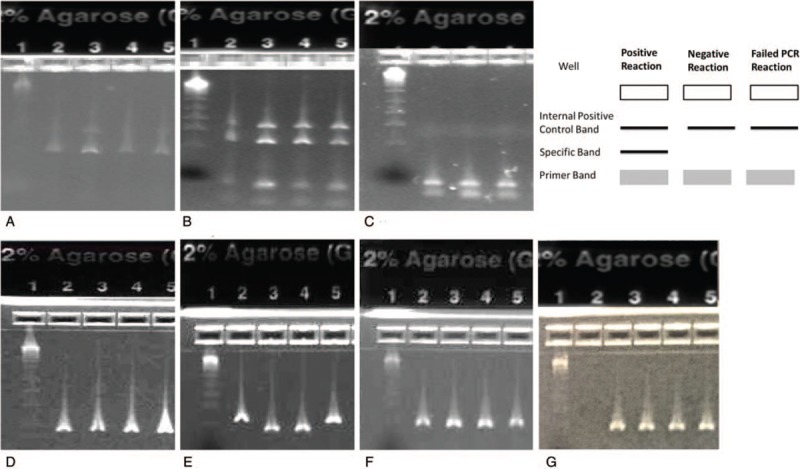
E-Gel Verification of HLA Class1-A, -B, -C Loci, & HLA Class II -DRB1, -DRB345, -DQA1/B1, and -DPA1/B1 Typing. This is a picture of a 2% agarose E-Gel electrophoresis showing the pattern of HLA class I and class II specific PCR genotyping products. E-Gel shows the band pattern for a) HLA Class I-A, b) HLA-B, and c) HLA-C. d) HLA Class II-DRB1, e) HLA-DRB345, f) HLA-DQA1/B1, g) and HLA -DPA1/B1. The DNA ladder is in the first lane, while wells 2-5 contain representative PCR products from patients. Control sample is in h) lane 2 indicating that genomic DNA of each sample is successfully amplified. The right upper illustration shows the interpretation pattern. Each PCR reaction includes a unique set of primers that are intended to matched with a single allele or group of alleles and produce a PCR product with a particular known size.

HLA typing was done using LABType SSO kits from One Lambda-Thermofisher Scientific and Luminex 100/200. Briefly, polymerase chain reaction (PCR) was used to amplify HLA class I: (HLA-A, -B, and -C) and class II: (HLA-DRB1, -DRB345, -DQA1/B1, and -DPA1/B1) using C1000 BioRad thermal cycler. PCR samples were loaded onto a 2% agarose gel to verify the amplified product (Fig. 1). The PCR products then underwent denaturation and hybridization with Luminex microspheres coated with specific probes for HLA- A, -B, -C, -DRB1, -DRB345, -DQA1/B1, and -DPA1/B1 complementary sequence, followed by labeling with an R-PE conjugated streptavidin secondary antibody. The hybridized product was detected using Luminex 100/200 instrument and analyzed using HLA Fusion 3.0-HLA by One Lambda.

### 2.4 Statistical analysis

Non-parametric analysis of HLA alleles between 2 groups was done using Chi Square (χ^2^) test as appropriate. The data for the alleles were shown as frequency (n) and percentages (%). Mann–Whitney *U* test was used to test for continuous variables and data was shown as mean ± standard deviation (SD). Multivariate regression analysis was presented as [odds ratio (OR); 95% confidence interval (CI)] after adjusting for significant demographic, clinical and biochemical confounders. Outcomes were disease subtypes and histological features. A *P* value of ≤.05 was considered to be significant. To limit the chance of over-fitting, the HLA-alleles were preselected at the univariate stage (*P* <.10) and only predictors with *P* <.05 were left in the models after bidirectional stepwise selection. All statistical analyses were performed using statistical software JMP 9.2 (SAS Institute, Cary, NC).

## 3 Results

### 3.1 Demographic and clinical characteristics of NAFLD patients and controls

Clinical and demographic data for both cases and controls are summarized in Table 1.

**Table 1:**
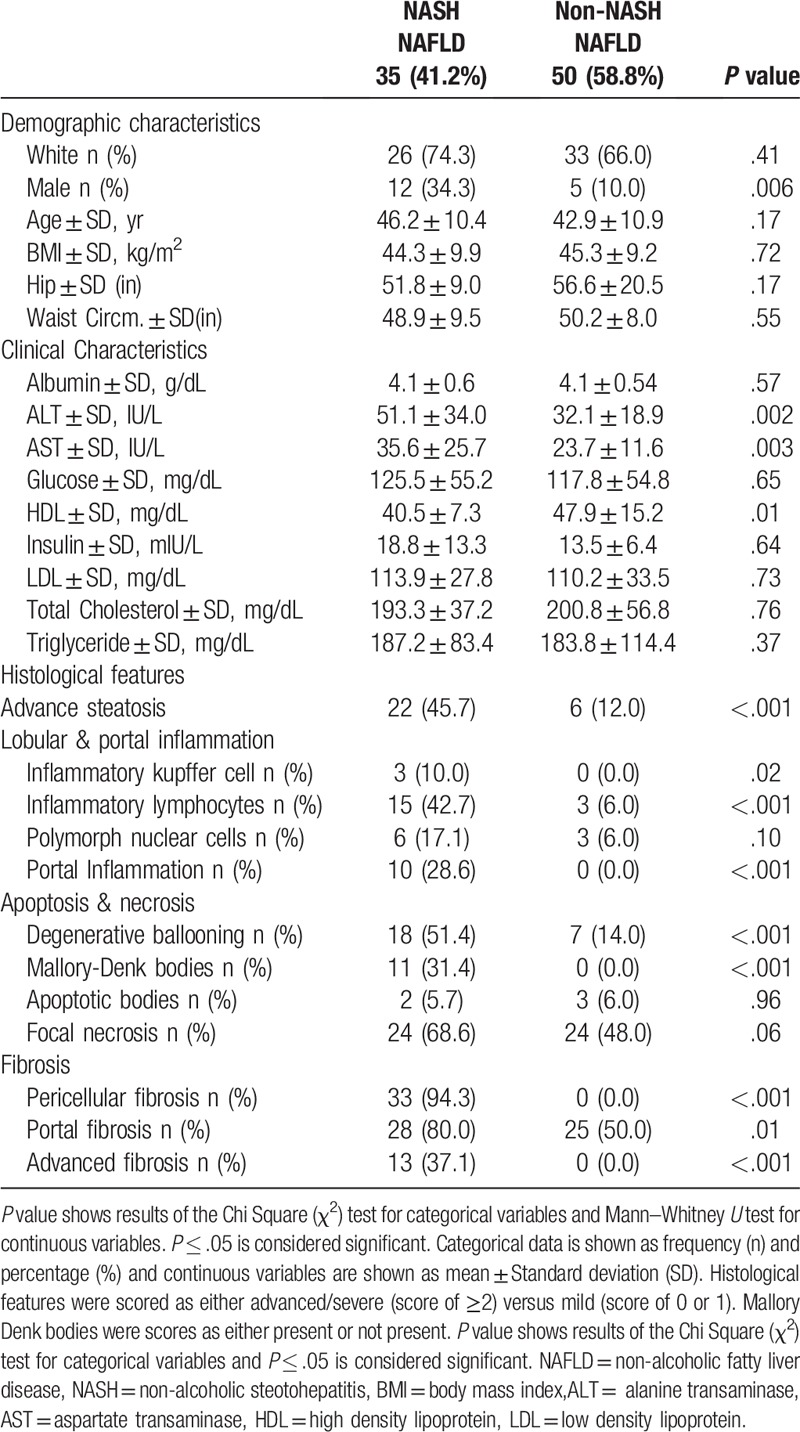
Demographic, clinical, and histological characteristics of NAFLD patients.

As expected, NAFLD had higher body mass index (BMI) and other components of metabolic syndrome. Among NAFLD, patients with NASH had significantly higher levels of alanine transaminase (ALT) [51.1 ± 34.0] and aspartate transaminase (AST) [35.6 ± 25.7] than non-NASH NAFLD patients (n=50), ALT [32.1 ± 18.9, *P* = .002] and AST [23.7 ± 11.6, *P* = .003]. The non-NASH NAFLD patients had significantly higher levels of high-density lipoprotein (HDL) than NASH patients [47.9 ± 15.2 vs 40.5 ± 7.3, *P* = .01] (Table 1).

Histological Features of NASH NAFLD and non-NASH NAFLD patients are also summarized in Table 1. Again, as expected NASH had significantly more lobular and portal inflammation than the non-NASH NAFLD group.

### 3.2 Association of HLA class I (HLA-A, -B, and -C) alleles with NAFLD

Univariate analysis revealed that HLA-A∗25 was significantly less frequent in NAFLD patients (1.2%) versus healthy controls (10.9%), *P* = .01. Similarly, HLA-B∗27 was less frequent in NAFLD (5.9%) versus controls (14.6%), *P* = .08. On the other hand, HLA-B∗51 and Bw65 were found only in the patients with NAFLD, (11.8%), *P* = .008 and (5.9%), *P* = .06 respectively (Table 2).

**Table 2:**
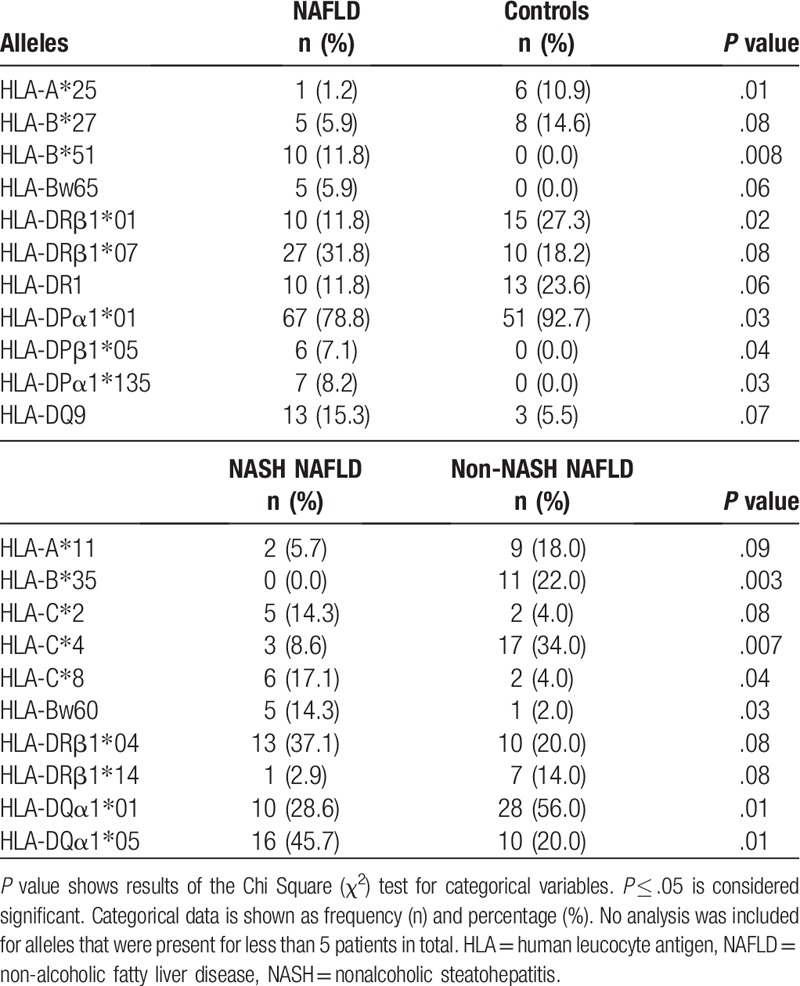
HLA class I and II alleles frequency, percentages, and association with NAFLD, controls, NASH NAFLD, and non-NASH NAFLD.

After adjusting for age, gender, race, diabetes, hyperlipidemia, hypertension and all significant alleles shown by univariate analysis, multivariate analysis showed no independent association with NAFLD.

### 3.3 Association of HLA class II (HLA-DR, -DQ, and -DP) alleles with NAFLD

Univariate analysis showed that the alleles that were less frequent in NAFLD included HLA-DRB1∗01 (11.8%) versus controls (27.3%), *P* = .02 and HLA-DPA1∗01 (78.8%) versus controls (92.7%), *P* = .03. No alleles in the DR3 locus were associated with NAFLD (Table 2).

On the other hand, in multivariate analysis, HLA-DRB1∗07 was independently associated with higher risk for NAFLD [OR = 3.21 (CI: 1.05–9.79), *P* = .04] after adjusting for age, gender, race, diabetes, hyperlipidemia, hypertension, and all significant alleles.

### 3.4 Association of HLA class I (HLA-A, -B, and -C) alleles with NASH

In the B locus, HLA-B∗35 was absent in biopsy-proven NASH (0.0%) while it was found in 22.0% of non-NASH NAFLD patients, *P* = .003. Serologically, HLA-Bw60 was significantly higher in NASH NAFLD (14.3%) versus non-NASH NAFLD (2.0%), *P* = .03. Similarly, HLA-C∗8 was significantly higher in NASH NAFLD (17.1%) than non-NASH NAFLD patients (4.0%), *P* = .04. In contrast to these findings, HLA-C∗4 was found to be significantly higher in non-NASH NAFLD patients (34.0%) as compared to NASH NAFLD patients (8.6%), *P* = .007 (Table 2).

**Figure 2:**
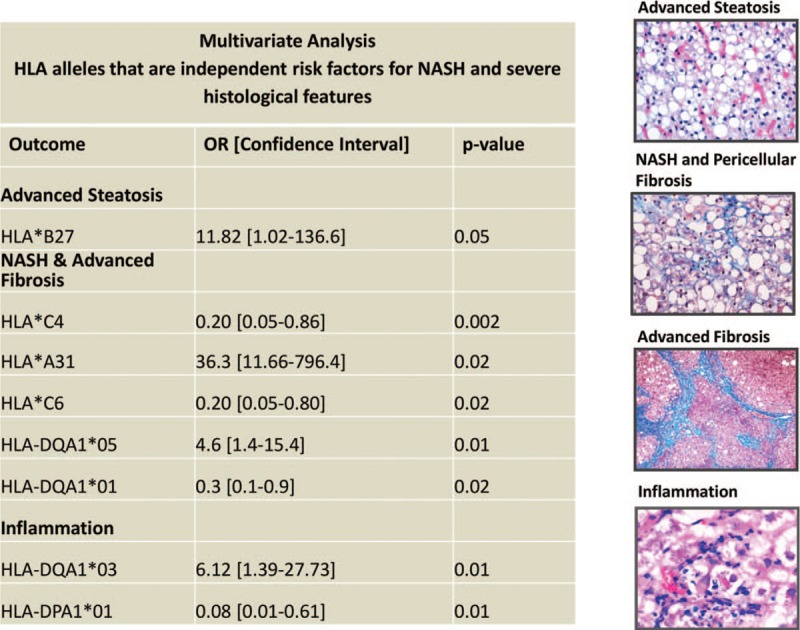
HLA Class I and II Alleles Are Independently Associated with Histological Features of NAFLD. Representative immunohistochemical images showing advanced steatosis (top), NASH/pericellular fibrosis and advanced fibrosis (bridging fibrosis and cirrhosis) (middle) and inflammation (bottom) in patients with NAFLD. Sections showing steatosis are stained with hematoxylin and eosin sections showing fibrosis are stained with Masson Trichrome to stain for collagen deposition. Table showing Multivariate analysis; HLA-B∗27 was independently and positively associated with advanced steatosis. HLA-C∗4, HLA-A∗31, HLA-C∗6, HLA-DQA1∗05 and HLA-DQA1∗01 were independently associated with NASH and advanced fibrosis after adjusting for confounders. HLA-DQA1∗03 and HLA-DPA1∗01 were independently associated with inflammation.

In the multivariate model, and after adjusting for age, gender, race, diabetes, hyperlipidemia, hypertension and all significant alleles shown by univariate analysis, HLA-C∗4 was found to be associated with lower risk for NASH [OR = 0.2 (CI: 0.05–0.86), *P* = .002] (Fig. 2).

### 3.5 Association of HLA class II (HLA-DR, -DQ, and -DP) alleles with NASH

Univariate analysis showed that within the DQ locus, HLA-DQA1∗05 was significantly more frequent in patients with NASH NAFLD (45.7%) versus non-NASH NAFLD (20.0%), *P* = .01. In contrast, HLA-DQA1∗01 was more frequent in the non-NASH NAFLD patients (56.0%) versus NASH NAFLD patients (28.6%), *P* = .01. HLA-DRB3 was associated with NASH NAFLD (27.3%) versus non-NASH NAFLD (9.1%), *P* = .03 (Table 2).

In the multivariate model, and after adjusting for age, gender, race, diabetes, hyperlipidemia, hypertension, and all significant alleles shown by univariate analysis, HLA-DQA1∗01 [OR = 0.3 (CI: 0.1–0.9), *P* = .02] was independently associated with lower risk for NASH NAFLD while HLA-DRB∗03 [OR = 3.75 (CI: 1.04–13.5), *P* = .04] was independently associated with higher risk for NASH NAFLD (Fig. 2). Male gender [OR = 5.2 (CI: 1.33–20.39), *P* = .03] was 1 confounder that was also independently associated with higher risk for NASH.

### 3.6 Association of HLA class I (HLA-A, -B, and -C) alleles with histological features of NAFLD

The univariate associations of HLA class I with different histological features of NAFLD are summarized in Table 3.

**Table 3:**
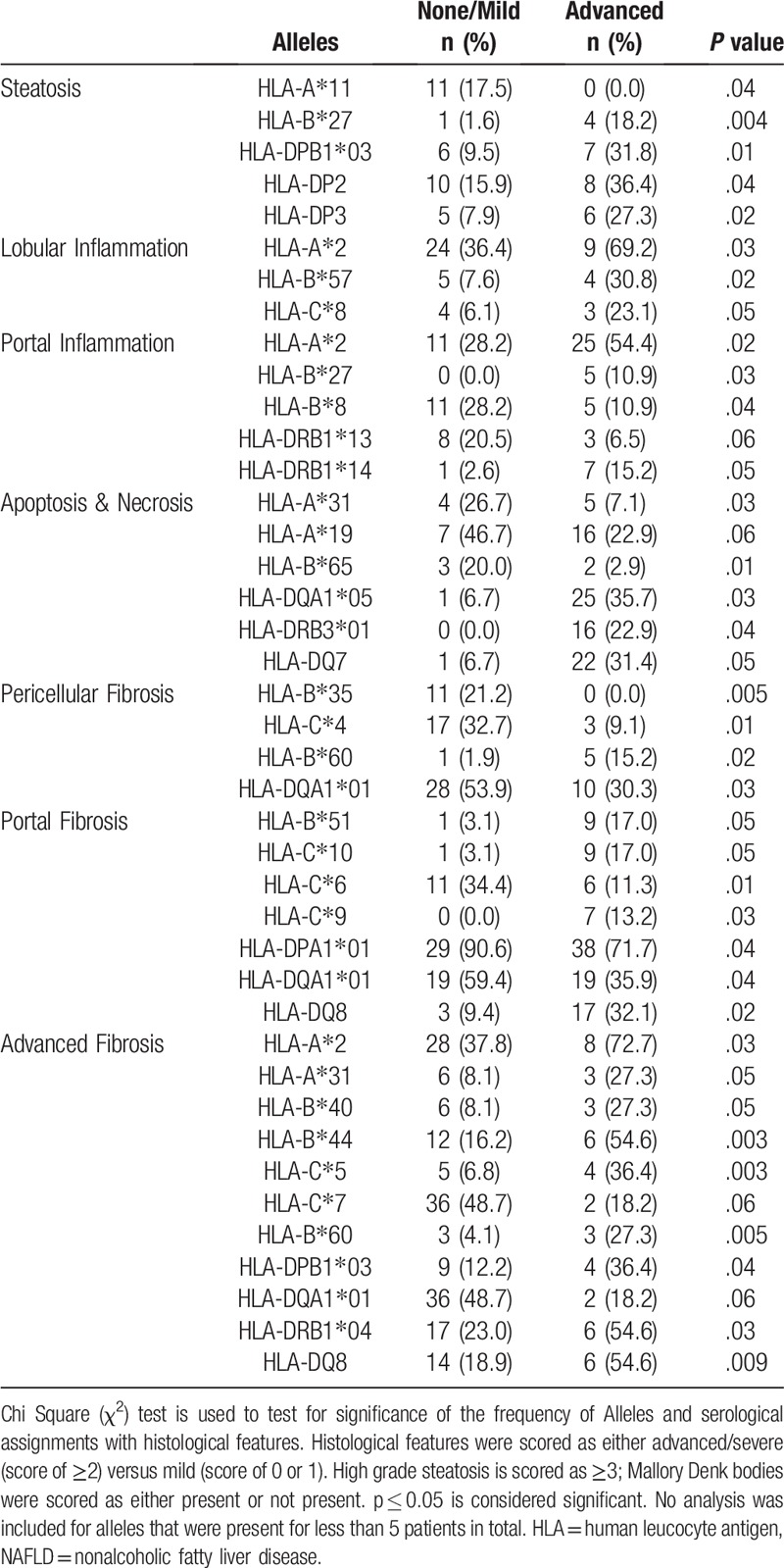
Univariate analysis of HLA class I and II alleles and different histological features of NAFLD.

The multivariate models adjusted for age, gender, race, diabetes, hyperlipidemia, hypertension, and all significant alleles shown by univariate analysis, revealed that HLA-B∗27 [OR = 11.7 (CI: 1.1–131.3), *P* = .05] was independently associated with high risk for high-grade steatosis. In addition, serological HLA-Bw65 [OR = 0.03 (CI: 0.00–0.41), *P* = .008] was found to be negatively associated with apoptosis and necrosis. In contrast, HLA-C∗6 [OR = 0.2 (CI: 0.05–0.8), *P* = .01] was independently protective against portal fibrosis and HLA-C∗4 [OR = 0.2 (CI: 0.05–0.8), *P* = .03] was protective against pericellular fibrosis. Finally, HLA-A∗31 [OR = 36.1 (CI: 1.7–786.1), *P* = .02] was independently associated with advanced fibrosis (Fig. 2).

### 3.7 Association of HLA class II (HLA-DR, -DQ, and -DP) alleles with histological features of NAFLD

HLA DQA1∗01 was independently and significantly associated with lower risk for NASH NAFLD, *P* = .02. This finding was confirmed when comparing the results to the histological features associated with NASH. As expected, this allele was also independently associated with lower risk for portal fibrosis [OR = 0.31 (CI: 0.11–0.91), *P* = .03] and pericellular fibrosis [OR = 0.31 (CI: 0.11–0.89), *P* = .03]. HLA-DQA1∗05 was associated with NASH NAFLD, *P* = .01 (Table 2), and this finding was confirmed by its association with apoptosis and necrosis, *P* = .03 (Table 3).

Furthermore, in the multivariate analysis of alleles and histological features, HLA-DP2 [OR = 3.4 (CI: 1.0–11.9), *P* = .05] was found to be independently associated with higher risk of high-grade steatosis. HLA-DP3 [OR = 11.6 (CI: 1.2–112.5), *P* = .03] and HLA-DQ8 [OR = 33.2 (CI: 1.2–895.8), *P* = .04] were independently associated with advanced fibrosis. Multiple genes were found to be independently linked to hepatic inflammation. HLA-DRB1∗14 [OR = 15.9 (CI: 1.2–204.3), *P* = .03] was found to be independently associated with portal inflammation. HLA-DQA1∗03 [OR = 6.21 (CI: 1.4–27.7), *P* = .02] was independently linked to presence of Kupffer cells in the liver. HLA-DPA1∗01 [OR = 0.08 (CI: 0.01–0.610), *P* = .01] and -DP4 [OR = 0.09 (CI: 0.01–0.87), *P* = .04] were independently associated with lower risk for polymorphonuclear cell inflammation (Fig. 2).

### 3.8 Association of HLA class I (HLA-A, B, and C) alleles and HLA class II (HLA-DR, -DQ, and -DP) alleles with clinical covariates of the metabolic syndrome in NAFLD patients

Alleles within the A loci were associated with diabetes and hyperlipidemia in this NAFLD cohort, while Alleles in the B loci were associated with hypertension. HLA-A∗2 [OR = 0.3 (CI: 0.1–0.7), *P* = .01] was found to be significantly and negatively associated with NAFLD in patients diagnosed with diabetes. Inversely, HLA-A∗19 [OR = 3.4 (CI: 1.3–9.3), *P* = .01] was positively correlated with diabetes. HLA-A∗9 [OR = 3.2 (CI: 1.0–9.7), *P* = .04] was significantly associated with hyperlipidemia. HLA-B∗15 [OR = 3.9 (CI: 1.0–15.0), *P* = .05] was associated with hypertension, while HLA-B∗35 [OR = 0.2 (CI: 0.06–0.1), *P* = .05] was found to be negatively associated with hypertension.

When studying the association of the HLA class II alleles with NAFLD comorbidities, the covariate analysis revealed that HLA DRB1∗1 [OR = 0.16 (CI: 0.03–0.82), *P* = .03] had a significantly negative association with hypertension. HLA class II alleles were not significantly associated with either diabetes or hyperlipidemia in this NAFLD cohort.

## 4 Discussion

This is an extensive assessment of HLA type I and II in patients with biopsy-proven NAFLD. Increasing evidence suggests that NAFLD is a complex disease involving both genetic and environmental factors.^[11–14]^ In the liver, hepatocytes, Kupffer cells, sinusoidal cells, and endothelial cells express MHC class II molecules that are encoded by HLA genes.^[15–17]^ HLA loci are highly polymorphic and may confer greater risk for NASH and NASH-related cirrhosis.^[2,4,5]^ In the present study, we have examined for genetic predisposition for NASH and advanced fibrosis within the HLA loci among biopsy-proven NAFLD and its subtypes.

Our data show that HLA-Bw65 was found in patients with NAFLD but not in controls without NAFLD. This finding is consistent with another recent study indicating that HLA-B∗65 may be an independent risk factor for NAFLD.^[2]^ Additionally, we found that HLA-Bw60 was significantly more frequent in patients with NASH and was linked to pericellular as well as advanced fibrosis. Moreover, HLA-B∗40 and HLA-B∗35 have been linked to cirrhosis in alcoholics.^[3,4]^ Similarly, in our study, HLA-B∗40 and HLA-B∗44 were correlated with advanced fibrosis. However, HLA-B∗35 was only present in patients who did not have pericellular fibrosis. Taken together, this data may indicate that the B loci may share similar but specific pathogenic pathways in fibrosis in alcoholic and non-alcoholic fatty liver diseases.

Our results also showed a negative independent association between HLA-C∗4 and pericellular fibrosis, a hallmark diagnostic feature of histologic NASH. Since Cw∗04 is a ligand for the killer immunoglobulin-like receptors on natural killer cells, these cells may be involved in protection against fibrosis as well as recovery from HCV infection.^[18]^ Furthermore, we hypothesize that Cw∗4 is a molecule that may play a role in the lipid pathway of antigen presentation, a common pathway for both diseases as well as initiation of collagen formation.

Within HLA class II loci, HLA-DRB1∗07 was associated with higher risk for NAFLD and HLA-DRB∗03 was independently associated with higher risk for NASH. Similarly, the same loci have been implicated in other immune-mediated diseases.^[19–21]^ Within the DQ loci, HLA-DQA1∗01 was associated with lower risk for NASH. Interestingly, DQA1∗01 has been linked to protection against HBV associated cirrhosis.^[22]^ Furthermore, HLA- DPB1∗03 which was independently associated with advanced fibrosis in our study, has been found to be strongly associated with risk of persistent infection with HBV infection.^[23]^ Therefore, alleles within the DQ loci may have a pathogenic role and alleles within the DP loci may have a protective role.

Our data showed that HLA-A∗2 was significantly associated with lobular and portal inflammation, advanced fibrosis, as well as with NAFLD patients who have diabetes but not hyperlipidemia. The same allele was reported to be an independent risk factor for drug-induced liver injury^[24]^ as well as a predictor of the response to treatment to HCV.^[25]^ Therefore, it is possible that HLA-A∗2 affects NAFLD immunopathogenesis by preparation of molecules for antigen presentation that will initiate an immunological environment that is most favorable for induction of liver cell injury. The HLA-A∗2 molecule has long gained attention as a potential immunotherapeutic molecule not only within the liver context but in the field of other chronic diseases including type I diabetes.^[26–31]^ Furthermore, in NAFLD patients, HLA-DRB1∗14 was independently associated with portal inflammation. Interestingly this allele was shown to be higher in patients with HCC.^[8]^ This may indicate that patients with NASH who are HLA-DRB1∗14 share common inflammatory pathways with patients with HCC and therefore may be at a higher risk for development of HCC.

Taken together this data suggest that HLA antigens are key players in shaping the cellular immune response in both health and disease and share common inflammatory pathogenic pathways that may be critical for disease progression in chronic liver disease including NAFLD.^[32,33]^ Therefore, all this data may indicate that some host factors associated with NAFLD might be linked to alleles within the HLA molecule that regulates immune-inflammatory responses.

Despite the extensive nature of the HLA assessment in this study, there are some limitations. In fact, our data does not provide evidence for direct causative relation between HLA and NASH-related outcomes. The present study is an investigative analysis of NASH and liver fibrosis and needs validation in a larger group of patients with NASH-related fibrosis. In fact, given the number of assessed parameters and limited sample size, we could not rule out the risk of over-fitting for the presented multivariate models. Nevertheless, the strengths of this study were the selection of the very well-defined biopsy-proven NAFLD patients, and extensive genotyping in medium to high resolution for both HLA class I and II to uncover markers relevant to liver fibrosis.

In summary, our findings point to the involvement of new pathways in the pathogenesis of NAFLD and provide more insight into the genetic basis of NAFLD. In particular, several HLA class I and II molecules were found to be associated with either protection against or susceptibility to NAFLD, supporting the importance of the immune response in the outcomes of NASH and fibrosis. Furthermore, NAFLD is a heterogeneous disease comprising different biological subtypes and this study is a valuable addition in identifying patients with favorable outcomes for different therapeutic options for NASH. However, cellular immunity needs to be further studied within the NAFLD population.

## Author contributions

**Data curation:** Azza Karrar, Zahra Younoszai, Thomas Jeffers, Fanny Monge, Zachary Goodman, Zobair M. Younossi.

**Formal analysis:** Azza Karrar, Li Zheng, Otgonsuren Munkhzul, Sharon Sharon Hunt, Zobair M. Younossi.

**Investigation:** Azza Karrar, Zobair M. Younossi.

**Methodology:** Azza Karrar, Siddharth Hariharan, Yousef Fazel, Ali Moosvi, Mohamad Houry, Zachary Goodman.

**Writing – original draft:** Azza Karrar, Siddharth Hariharan, Ali Moosvi, Mohamad Houry.

**Writing – review & editing:** Zobair M. Younossi.
